# Psychometric properties of the Spanish version of the Patient Health Questionnaire in Mexican adolescents

**DOI:** 10.3389/fpsyg.2025.1634804

**Published:** 2025-10-08

**Authors:** Christian Díaz de León Castañeda, Lina Díaz-Castro

**Affiliations:** ^1^Investigadores por México, Secretaría de Ciencia, Humanidades, Tecnología e Innovación (SECIHTI), Mexico City, Mexico; ^2^Departamento: Dirección de Investigaciones Epidemiológicas y Psicosociales, Instituto Nacional de Psiquiatría Ramón de la Fuente Muñiz, Mexico City, Mexico

**Keywords:** depression, adolescent, psychometrics, confirmatory factor analysis, reliability

## Abstract

**Introduction:**

The present study aimed to contribute to the analysis of the psychometric properties of the Patient Health Questionnaire (PHQ-9) in a sample of adolescents residing in San Luis Potosí (SLP), Mexico.

**Methods:**

768 adolescents from SLP, Mexico, participated in the study. Confirmatory factor analysis (CFA) was conducted to test the one and two-factor models, taking into account the ordinal nature of the items in the model estimation method. Internal consistency was evaluated using the Coefficient alpha (α) and Coefficient omega (ω). Invariance measurement by sex was tested. Also, the relationship with the General Anxiety Disorder (GAD-7 scale) was tested.

**Results:**

The CFA analyses indicated that both models, the one-factor model and the two-factor model, had a very good fit. The single factor and the two factors of the models demonstrated acceptable internal consistency (Coefficient alpha ranging from 0.779 to 0.896). Both models demonstrated measurement invariance by sex up to the *Strong* level. Additionally, a high Pearson correlation was found between the PHQ-9 and GAD-7 total scores (r = 0.812).

**Conclusion:**

It is concluded that the PHQ-9 scale presents good psychometric properties for the Mexican adolescent population.

## 1 Introduction

Adolescent depression constitutes a major public health challenge in Mexico and Latin America, especially in contexts marked by social vulnerability and marginalization. Adolescents exposed to adverse circumstances such as poverty, violence, lack of family support, and discrimination show a higher prevalence of depressive symptoms and self-injurious or suicidal behaviors (Marín-Tejeda et al., 2021; Pérez-Amezcua et al., 2010). These symptoms commonly include persistent sadness, irritability, anhedonia, sleep and appetite disturbances, low self-esteem, emotional regulation difficulties, and, in severe cases, suicidal ideation or attempts (Valadez-Figueroa et al., 2019; García Lara et al., 2018).

Scientific literature demonstrates a strong relationship between depressive symptoms in adolescents and risk factors such as impulsivity, family violence, sexual abuse, body dissatisfaction, substance use, and poor communication with parents (Guadarrama Guadarrama et al., 2014; Espinoza-Gómez et al., 2010; Pérez-Amezcua et al., 2010). For example, a national study found that nearly half of upper secondary students reported at least one symptom of suicidal ideation, with depressive symptoms being one of the main predictors (Pérez-Amezcua et al., 2010). Furthermore, adolescent girls, who are often more exposed to risk factors like violence, show higher rates of depressive symptoms and suicidal ideation (González-Forteza et al., 1998).

Early and accurate detection of depressive symptoms is essential to prevent serious consequences such as suicide, school dropout, and deterioration of family and social relationships (Borges et al., 2019). To achieve this, it is crucial to use robust, validated, and culturally relevant psychometric instruments. Among those used in Latin American research are the Center for Epidemiologic Studies Depression Scale (CES-D), Beck’s Suicidal Ideation Scale, and the Patient Health Questionnaire-9 (PHQ-9) (Terrones-González et al., 2012; González-Forteza et al., 2011). The PHQ-9, in particular, is widely recommended due to its brevity, ease of use, and strong psychometric validity for screening, diagnosing, and monitoring the presence and severity of depressive symptoms, including its use in adolescents (Kroenke et al., 2001; Kroenke and Spitzer, 2002).

The PHQ-9 scale is based on the symptoms of the DSM-IV diagnostic criteria for major depressive disorder (American Psychiatric Association [APA], 2000) and is considered generic; that is, it can be used in different demographic groups (sex, age group, health condition, etc.). The PHQ-9 is a 9-item scale and is self-reported; persons should take into account 2 weeks prior to the evaluation to respond to the items. A shorter version, PHQ-8, has been proposed to avoid ethical implications that could have an affirmative response to the last item of PHQ-9 (about having suicidal ideation) (Kroenke et al., 2009).

The PHQ-9 scale and PHQ-8 scale have been translated to several idioms, showing good performance in validity and reliability (Arias de la Torre et al., 2023; Lamela et al., 2020). Regarding the factorial structure of the PHQ-9, it has been probed and confirmed its unidimensional (one-factor) structure mainly, but other structures have been proposed, such as two-dimensional (two-factor) structures, mainly one that recognizes a “Cognitive/Affective” factor and a “Somatic” factor; also, bifactorial models have been proposed (Doi et al., 2018; Petersen et al., 2015). However, there have been discrepancies about what structure is better, the one-factor, two-factor, or bifactor structures (Lamela et al., 2020), although this could be difficult to define since the scale is of generic use, so it could have different behavior or structure in different populations (different age groups, and health conditions such as clinical, psychiatric, and general populations).

Also, some methodological limitations of the published studies have been identified, mainly regarding the validity and reliability of versions adapted to other languages in the context of Low to Middle-Income Countries (LMIC) (Carroll et al., 2020). The Spanish version of the PHQ-9 scale has been tested in some countries, such as Spain, Peru, Ecuador, Puerto Rico, Chile, Colombia, and Mexico, in different populations. However, as mentioned before, regarding the definition of a factorial structure, different results have been obtained that could be related to the application of the scale in different populations. A previous study performed in Mexico with a high sample size of Mexican teachers (non-clinical sample) supported only the one-factor model (Familiar et al., 2015), and another study performed in adults (Arrieta et al., 2017) also concluded the same. Also, other studies performed in this language have supported the one-factor model (Fonseca-Pedrero et al., 2023; López-Torres et al., 2022; Saldivia et al., 2019; Villarreal-Zegarra et al., 2019); however, other studies performed with different populations have supported two-factor or bi-factor models (Huarcaya-Victoria et al., 2020; López-Guerra et al., 2022; Quiñonez-Freire et al., 2021).

In the context of this panorama, it is identified that little research has been done on the performance of the PHQ-9 scale in adolescents. The present study aims to contribute to the study of the psychometric properties of the Spanish version of the PHQ-9 scale in adolescents.

## 2 Methods

### 2.1 Design

A cross-sectional study was conducted between April and September 2023 in communities within the municipality of Ciudad Fernández, in the State of San Luis Potosí, Mexico. Data were collected using questionnaires as part of an ongoing research project (Project number S.736, funded by the Gonzalo Río Arronte Foundation I.A.P.) to assess the prevalence of depressive symptoms in adolescents.

### 2.2 Setting

San Luis Potosí, at the 2020 national census, had a total population of 2,822,255, approximately 17%, around 480,000 individuals, had between 15 and 24 years old. Besides, 8.60% of the population speaks an indigenous language (one of the Mexican states with the highest number of indigenous language speakers), of which, 4.32% does not speak Spanish (Instituto Nacional de Estadística y Geografía [National Institute of Statistics and Geography] (INEGI), 2020).

Ciudad Fernández is a municipality in the middle zone of San Luis Potosí. It includes the municipal seat, 13 “ejidos” (communal agricultural lands), and 19 ranches. According to the 2020 census, the municipality had 48,106 inhabitants. Ciudad Fernández is the most populated locality, concentrating 36,275 residents. Despite being a territory characterized by rural and indigenous communities, almost the entire population speaks Spanish, and less than 1% of the population speaks an indigenous language (being bilingual) (Instituto Nacional de Estadística y Geografía [National Institute of Statistics and Geography] (INEGI), 2020).

#### 2.2.1 Participants

A total of 786 fulfilled the questionnaire correctly. Participants were adolescents studying high-school or a bachelor’s degree and living in the Municipality of Ciudad Fernández, San Luis Potosí, Mexico, during the study period. Table 1 presents the main socio-demographic characteristics of the participants.

**Table 1:** Socio-demographic characteristics of participants (*n* = 768).^a^

Variables		*n*	%
Adolescents			
Academic degree, (*n*, %)	High-School	702	91.4
	Bachelor	66	8.6
Sex (*n*, %)	Woman	456	59.8
	Man	306	40.2
Age in years (M, SD)		16.8	2.4
Speak an indigenous language (*n*, %)	No	724	99.5
	Yes	4	0.5
Parent or caregivers			
Sex (*n*, %)	Woman	439	65.8
	Man	228	34.2
Age (M, SD)		54.6	23.1
Schooling in completed years of study (M, SD)		11.3	1.5
Home			
Monthly Household Income, Mexican pesos (M, SD)		10,973.90	11,894.10
Monthly Household Income per person, Mexican pesos (M, SD)		3,037.40	3,273.40

a M = Mean, SD = Standard deviation.

### 2.3 Instruments

A structured questionnaire was designed to include items exploring the socio-demographic variables pertaining to the participants and the items of the following scales:

(a) *Patient Health Questionnaire-9* (PHQ-9) (Kroenke et al., 2001; Kroenke and Spitzer, 2002). This is a generic 9-item scale (self-reported) for screening, diagnosing, and monitoring depression. Persons should take into account 2 weeks prior to the evaluation to respond to the items. Items have four response levels: “not at all,” “several days,” “more than half the days,” and “nearly every day” (0–3 scores). The total score ranges from 0 to 27. According to the APA, the cut-off points of 5, 10, 15, and 20 represent different levels of depression (mild, moderate, moderately severe, and severe depression, respectively) (American Psychiatric Association [APA], 2000). This study used the Spanish version of this scale. Slight adaptations were made to some items on the scale to make them more understandable to adolescents.
(b) *Generalized Anxiety Disorder Scale* (GAD-7) (Spitzer et al., 2006). This is a generic 7-item scale (self-reported) that evaluates the symptoms and severity of anxiety and is based on the DSM-IV diagnostic criteria. Items have four response levels: “not at all,” “several days,” “more than half the days,” “nearly every day” (0–3 scores). The total score ranges from 0 to 21. This study used the Spanish version of this scale, and it showed good internal consistency (α = 0.880, ω = 0.881).

The questionnaire was delivered to the study participants in print form in order to be filled out by them. Support was provided to adolescents, if necessary, by a health promoter.

### 2.4 Data analysis

#### 2.4.1 Confirmatory factor analysis (CFA)

A CFA was conducted to test the one-factor and two-factor models of the PHQ-9 scale, as proposed by previous studies (Lamela et al., 2020). The application of the CFA used the estimation method WLSMV (weighted least square mean and variance), which takes into account the ordinal nature of the items (Brown, 2015). Adjustment indices were obtained as follows: statistical chi-square (χ^2^); comparative fit index (CFI, acceptable adjustment criterion >0.90); standardized root mean square residual (SRMR, acceptable adjustment criteria <0.080); and root mean square error of approximation (RMSEA, acceptable adjustment criteria <0.80) (Hu and Bentler, 1999).

#### 2.4.2 Measurement invariance

A measurement invariance (MI) analysis was carried out through a multigroup CFA of the one-factor and two-factor models. This analysis was carried out comparing by sex. Robust maximum likelihood (MLR) was used as the estimation method. The procedure that has been suggested in the literature related to the evaluation of four stages (Configural, Weak, Strong, and Strict) was followed; the last three through different levels of restrictions (equal factorial loads, equal intercepts, and variances of the errors) (Putnick and Bornstein, 2016). The use of the CFI and RMSEA adjustment indices was proposed with the same criteria mentioned before to evaluate the adjustment of the multigroup models. In addition, the changes in the indices between levels after the Configural invariance were evaluated, considering the tolerance suggested in the literature (ΔCFI ≤ 0.010 and ΔRMSEA ≤ 0.015).

#### 2.4.3 Reliability

As a reliability analysis approach, internal consistency was evaluated by calculating the α and ω coefficients for the models proposed.

#### 2.4.4 Relationship analysis with other variables

Pearson correlations were obtained for the relationships among the PHQ-9 and GAD-7 scores.

### 2.5 Software

The SPSS 27 program was used for database management and some descriptive analyses, as was the SPSS AMOS program for the generation of the path diagrams of the tested models. The RStudio program was used to perform the CFA analysis, using the psych, semTools, lavaan, and semPlot packages (Epskamp, 2015; Jorgensen et al., 2021; Revelle, 2020; Rosseel, 2012).

### 2.6 Ethical considerations

The research protocol applied by the present study was approved by the Ethical Committee of the National Institute of Psychiatry Ramon de la Fuente Muñiz (Register: CEI/C/034/2022, date: July 18, 2022).

## 3 Results

### 3.1 Descriptive analysis of the item scores

Table 2 presents the descriptive statistics: frequencies of category responses, means, standard deviations, skewness, and kurtosis for each item on the PHQ-9 scale. The nine items exhibit lightly skewed (right) and platykurtic distributions, mainly, except for item 9 (markedly right-skewed and leptokurtic). The general scores of the PHQ-19 scale also had a lightly right-skewed distribution, but near to be mesokurtic.

**Table 2:** Descriptive analysis of the PHQ-9 scale items responses (*n* = 768).^a^

	Item	Not at all (=0)	Several days (=1)	More than half of the days (=2)	Nearly every day (=3)	Statistics			
No.	[Spanish translation]	*n* (%)	*n* (%)	*n* (%)	*n* (%)	M	SD	g_1_	g_2_
1	Little interest or pleasure in doing things [Poco interés o placer en hacer cosas]	280 (36.5)	312 (40.6)	101 (13.2)	75 (9.8)	0.96	0.94	0.78	−0.25
2	Feeling down, depressed, or hopeless [Te has sentido decaído(a), deprimido(a) o sin esperanzas]	364 (47.4)	266 (34.6)	83 (10.8)	55 (7.2)	0.78	0.91	1.03	0.24
3	Trouble falling or staying asleep, or sleeping too much [Has tenido dificultad para quedarte o permanecer dormido(a), o has dormido demasiado]	288 (37.5)	261 (34.0)	97 (12.6)	122 (15.9)	1.07	1.06	0.66	−0.82
4	Feeling tired or having little energy [Te has sentido cansado(a) o con poca energía]	159 (20.7)	374 (48.7)	129 (16.8)	106 (13.8)	1.24	0.93	0.53	−0.52
5	Poor apetite or overeating [Sin apetito o has comido en exceso]	334 (43.5)	245 (31.9)	100 (13.0)	89 (11.6)	0.93	1.01	0.82	−0.48
6	Feeling bad about yourself—or that you are a failure of have let yourself or your family down [Te has sentido mal contigo mismo(a)—o que eres un fracaso o que has quedado mal contigo mismo(a) o con tu familia]	316 (41.1)	254 (33.1)	114 (14.8)	84 (10.9)	0.96	0.99	0.75	−0.54
7	Trouble concentrating on things, such as Reading the newspaper or watching television [Has tenido dificultad para concentrarte en ciertas actividades, tales como leer el periódico o ver la televisión]	324 (42.2)	287 (37.4)	82 (10.7)	75 (9.8)	0.88	0.95	0.92	−0.07
8	Moving or speaking so slowly that other people could have noticed. Or the oposite—being so figety or restless that you have been moving around a lot more than usual [Te has movido o hablado tan lento que otras personas podrían haberlo notado. O lo contrario—muy inquieto(a) o agitado(a) que has estado moviéndote mucho más de lo normal]	437 (56.9)	220 (28.6)	73 (9.5)	38 (4.9)	0.62	0.85	1.29	0.87
9	Thoughts that you would be better off dead, or of hurting yourself [Has pensado que estarías mejor muerto(a) o has pensado en lastimarte de alguna manera]	546 (71.1)	147 (19.1)	40 (5.2)	35 (4.6)	0.43	0.79	1.94	3.06
	PHQ-9 Total score					7.87	6.24	0.93	0.12

a M = Mean; SD = Standard deviation; g_1_ = Asymmetry coefficient; g_2_ = Kurtosis coefficient.

Table 3 presents Pearson’s correlations among the PHQ-9 items and total scores; as expected, all were statistically significant and higher than 0.300. Regarding the correlation between the two factors of the two-factor model, their correlation was 0.749 (*p* < 0.001).

**Table 3:** Pearson correlations among the PHQ-9 items and total score (*n* = 768).^a^

	1	2	3	4	5	6	7	8	9	PHQ-9 Total
Item 1	--									
Item 2	0.530**	--								
Item 3	0.476**	0.484**	--							
Item 4	0.522**	0.531**	0.541**	--						
Item 5	0.490**	0.563**	0.547**	0.533**	--					
Item 6	0.475**	0.642**	0.410**	0.518**	0.545**	--				
Item 7	0.474**	0.503**	0.439**	0.551**	0.494**	0.534**	--			
Item 8	0.375**	0.523**	0.390**	0.446**	0.505**	0.498**	0.518**	--		
Item 9	0.343**	0.580**	0.335**	0.368**	0.455**	0.542**	0.376**	0.457**	--	
PHQ-9 Total	0.709**	0.802**	0.710**	0.758**	0.780**	0.779**	0.738**	0.700**	0.655**	--

a Statistical significance levels: *p < 0.050; **p < 0.010; ***p < 0.001.

### 3.2 Confirmatory factor analysis (CFA)

**Figure 1:**
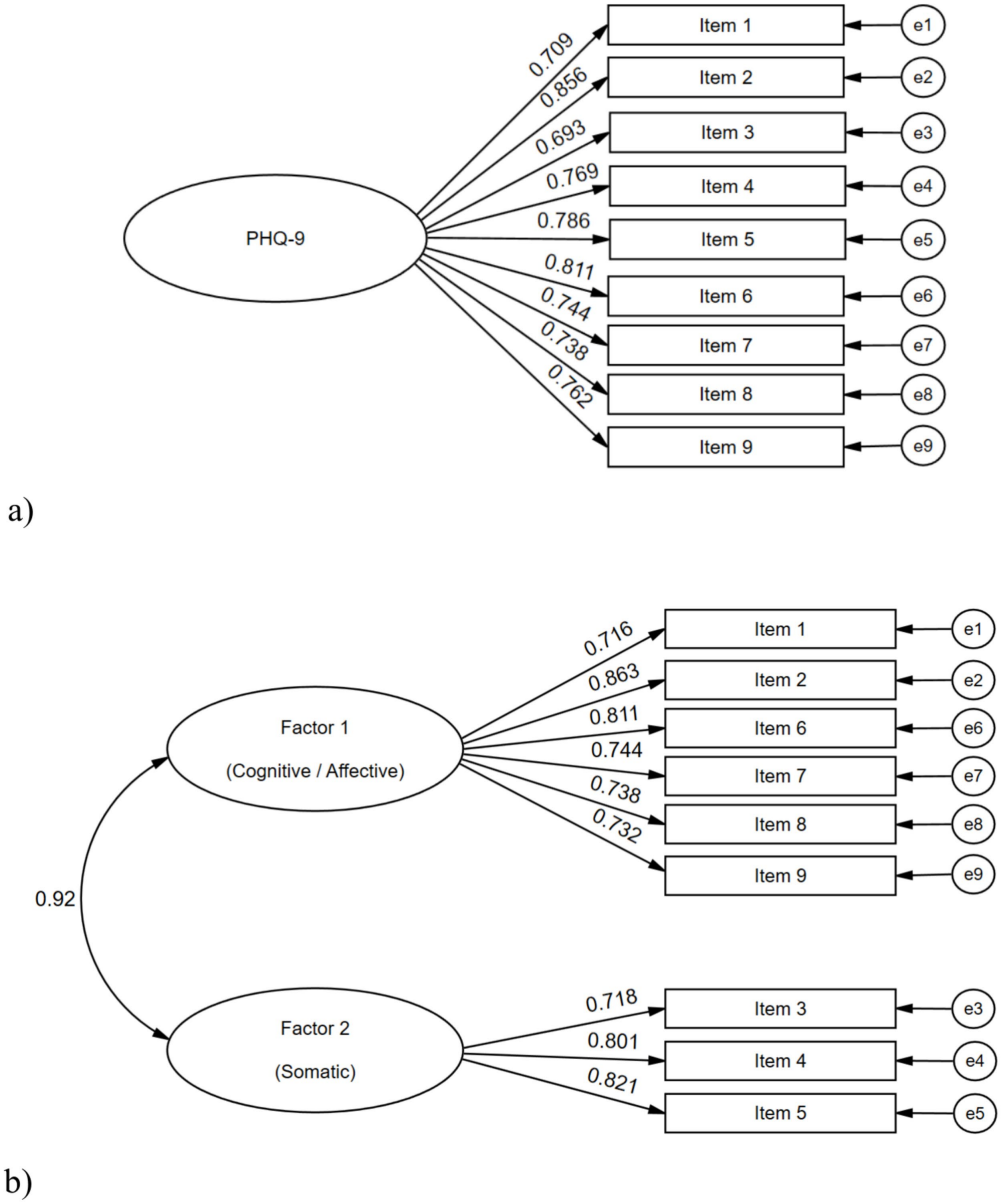
CFA results on the one-factor and two-factor models for the PHQ-9 scale, with all the participants (*n* = 768). **(A)** one-factor model; **(B)** two-factor model.

Figure 1 presents the factor loadings obtained for the two tested models. For the one-factor model, the factor loadings ranged from 0.693 to 0.856. For the two-factor model, the factor loadings ranged from 0.716 for Factor 1 and from 0.718 to 0.821 for Factor 2, while the correlation between these two factors was 0.920. All the loadings and factor covariances were statistically significant (*p* < 0.050).

The CFA results, as fit for the models analyzed, are presented in Table 4. The one-factor model had a good fit, although it did not meet the RMSEA criteria (χ^2^: 184.23, *p* < 0.001; CFI: 0.978; SRMR: 0.047; RMSEA: 0.087). The modification indices analysis suggested adding the covariance between the errors of items 2 and 9, which slightly improved the model fit (χ^2^: 162.57, *p* < 0.001; CFI: 0.981; SRMR: 0.044; RMSEA: 0.083).

**Table 4:** Results of the fit indices obtained from the CFA conducted on the PHQ-9 scale.^a^

Model	Error covariances	χ^2^	gl	CFI	SRMR	RMSEA
One-Factor	None	184.23***	27	0.978	0.047	0.087
	e2–e9	162.57***	26	0.981	0.044	0.083
Two-Factor	None	153.49***	26	0.982	0.043	0.080
	e2–e9	137.00***	25	0.985	0.040	0.076
	e2–e9/e4–e5	121.40***	24	0.987	0.038	0.073

a Statistical significance levels: *p < 0.050, **p < 0.010, ***p < 0.001.

The two-factor model had a slightly better fit than the one-factor model and accomplished all the criteria (χ^2^: 153.49, *p* < 0.001; CFI: 0.982; SRMR: 0.043; RMSEA: 0.080). The modification indices analysis suggested adding the covariance between the errors of items 2 and 9, which slightly improved the model fit (χ^2^: 137.00, *p* < 0.001; CFI: 0.985; SRMR: 0.040; RMSEA: 0.076). Also, the addition of another covariance between the errors of items 4 and 5 improved this model fit slightly.

### 3.3 Internal consistency

For the one-factor model, the Coefficient α was 0.894, and the Coefficient ω was 0.896. Regarding the two-factor model, for Factor 1, the Coefficient α was 0.852, and the Coefficient ω was 0.856; whereas for Factor 2, the Coefficient α was 0.778, and the Coefficient ω was 0.779.

### 3.4 Measurement invariance

The results of the measurement invariance evaluation are shown in Table 5. Regarding the analysis of MI by sex, it is observed that MI was demonstrated up to the Strong level for both models (without the release of parameters). However, the two-factor model had a better fit at the Configural level, in accordance with the result obtained in the CFA.

**Table 5:** Results for the measurement invariance analysis of the PHQ-9 scale (*n* = 768).^a^

Model	Invariance level	χ^2^	gl	CFI	ΔCFI	RMSEA	ΔRMSEA
One-Factor	Configural	166.78***	54	0.944	--	0.074	--
	Weak	176.39***	62	0.943	−0.001	0.070	−0.004
	Strong	191.03***	70	0.940	−0.003	0.067	−0.003
	Strict	229.57***	79	0.925	−0.015	0.071	+0.004
Two-Factor	Configural	144.27***	52	0.954	--	0.068	--
	Weak	152.39***	59	0.954	0	0.064	−0.004
	Strong	163.57***	66	0.952	−0.002	0.062	−0.002
	Strict	203.59***	75	0.936	−0.016	0.067	+0.005

a Statistical significance levels: *p < 0.050, **p < 0.010, ***p < 0.001.

### 3.5 Analysis of relationships with other variables

The Pearson correlation between the PHQ-9 and the GAD-7 total scores was 0.812 (*p* < 0.001). Regarding the correlations of the two factors of the two-factor model with the GAD-7 total score, they were: 0.799 (*p* < 0.001) for Factor 1, and 0.704 (*p* < 0.001) for Factor 2.

## 4 Discussion

The present study has provided evidence of the validity and reliability of the PHQ-9 scale, particularly the Spanish version and Mexican adaptation in its use for adolescents.

Regarding the internal structure analysis through CFA, this analysis primarily revealed both models, one-factor and two-factor, as being satisfactory via the CFA. However, the one-factor model slightly exceeded one of the indicators’ fit criteria (RMSEA > 0.80), which could be due to the simplicity of the model and few degrees of freedom, suggesting the need to try other models. Therefore, the two-factor model could be considered better, although the correlation between the factors was high, in accordance with previous studies of the Spanish version and in other languages (González-Blanch et al., 2018: Huarcaya-Victoria et al., 2020; Petersen et al., 2015; Quiñonez-Freire et al., 2021). This high correlation between factors seems to show that the two-factor model could be considered as inappropriate, so the use of the one-factor model in this age group could be suggested more.

Regarding the items’ performance, it should be noted that in both tested models, item 2 had the highest factorial load, showing that it is the one that could best discriminate or identify people with depressive symptoms. This result is consistent with previous studies conducted in other countries with versions in other languages (Arias de la Torre et al., 2023).

Other studies have found the bi-factor model to be more optimal in different age groups, in clinical and non-clinical samples (Doi et al., 2018; Lamela et al., 2020; López-Guerra et al., 2022), however, for the data collected in this study with the sample of adolescents studied, this model did not converge, since although a good fit was obtained, several of the factor loads were very small and close to zero, something similar to what was previously reported in a non-clinical sample of students (Huarcaya-Victoria et al., 2020).

It is worth mentioning that this is one of the few studies that have evaluated the adjustments of different models in the population, specifically of adolescents, since most of the published studies have worked with samples of adults over 18 years of age. Studies carried out specifically in adolescents have reported the fit of the one-factor model (Fonseca-Pedrero et al., 2023; López-Torres et al., 2022), so a contribution of this study is to demonstrate the fit of a two-factor model in this age group.

In relation to the analysis of measurement invariance, it was possible to demonstrate it by sex in both models tested, which is consistent with the literature, where this has also been demonstrated in different models and in versions of the PHQ-9 scale in various languages (Arias de la Torre et al., 2023), including the Spanish version, where, in addition, the measurement invariance related to other socio-demographic and socio-economic variables has been demonstrated (Villarreal-Zegarra et al., 2019).

To analyze the relationship with other variables, an analysis of convergent validity was performed with the GAD-7 scale, obtaining the expected results, namely a statistically significative Pearson correlation. This finding concurs with previous studies on the Spanish version of the PHQ-9 scale, which either found a significative correlation with GAD-7, and other related scales (Quiñonez-Freire et al., 2021).

Finally, among the strengths of the study, it is worth noting that the data analysis approaches recommended, particularly CFA, were employed, in accordance with the recent psychometric research literature. These approaches included the use of an estimator that accounts for the ordinal nature of the items (the WLSMV estimator) (Brown, 2015).

However, certain limitations of this study should be acknowledged. The PHQ-9 was applied to adolescents enrolled in high school and bachelor’s degree programs, living in rural and/or indigenous communities of San Luis Potosí, Mexico. Although primarily of indigenous origin, all participants speak Spanish, with fewer than 1% speaking also an indigenous language. This context may restrict the generalizability of the findings, but they remain applicable to similar communities. Another limitation of the study was the absence of convergent validity testing with other depression scales or related constructs.

Future research should consider carrying out construct, criterion, and predictive validity studies, as well as employing additional reliability assessment methods, such as test–retest procedures.

## 5 Conclusion

It is concluded that the Spanish version of the PHQ-9 scale, adapted in Mexico, retains strong psychometric properties and is a viable proposal for a scale that assesses depression in adolescents, particularly those belonging to rural and indigenous communities.

## Data Availability

The datasets presented in this article are not readily available because the dataset is not publicly available due to confidentiality and the sensitive nature of the information collected. Access to the data is restricted to protect the privacy of the adolescent participants. Requests to access the datasets should be directed to dralaindiaz.ld@gmail.com.
